# Ethyl 7-methyl-3-oxo-5-phenyl-2-(2,4,6-trimeth­oxy­benzyl­idene)-2,3-dihydro-5*H*-thia­zolo[3,2-*a*]pyrimidine-6-carboxyl­ate

**DOI:** 10.1107/S1600536812012354

**Published:** 2012-03-28

**Authors:** Noor Afshan Banu, V. Bheema Raju

**Affiliations:** a Department of Chemistry, KNS Institute of Technology, Bangalore 560 064, India; b Department of Chemistry, Dr Ambedkar Institute of Technology, Bangalore 560 056, India

## Abstract

In the title compound, C_26_H_26_N_2_O_6_S, the benzene ring is positioned axially to the thia­zolopyrimidine ring and bis­ects it with a dihedral angle of 80.94 (7)°. The pyrimidine ring adopts a flattened boat conformation. In the crystal, pairs of bifurcated C—H⋯O hydrogen bonds link the mol­ecules into chains along the *c* axis.

## Related literature

For the pharmacological activity of pyrimidine derivatives, see: Alam *et al.* (2010 ▶). For a related crystal structure, see: Chen *et al.* (2012 ▶).
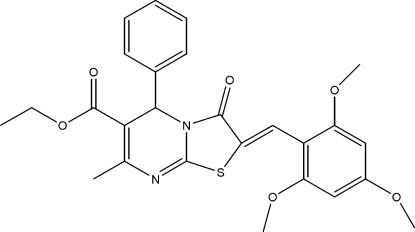


## Experimental

### 

#### Crystal data

C_26_H_26_N_2_O_6_S
*M* *_r_* = 494.55
Monoclinic, 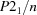

*a* = 7.5363 (19) Å
*b* = 18.178 (5) Å
*c* = 16.973 (4) Å
β = 94.465 (5)°
*V* = 2318.1 (10) Å^3^
*Z* = 4
Mo *K*α radiation
μ = 0.19 mm^−1^
*T* = 296 K
0.18 × 0.16 × 0.16 mm

#### Data collection

Bruker SMART APEX CCD detector diffractometer
Absorption correction: multi-scan (*SADABS*; Bruker, 1998 ▶) *T*_min_ = 0.967, *T*_max_ = 0.971
13918 measured reflections
5055 independent reflections
3500 reflections with *I* > 2σ(*I*)
*R*_int_ = 0.052

#### Refinement

*R*[*F* ^2^ > 2σ(*F* ^2^)] = 0.058
*wR*(*F* ^2^) = 0.168
*S* = 1.07
5055 reflections
321 parameters
H-atom parameters constrained
Δρ_max_ = 0.59 e Å^−3^
Δρ_min_ = −0.38 e Å^−3^

### 

Data collection: *SMART* (Bruker, 1998 ▶); cell refinement: *SAINT-Plus* (Bruker, 1998 ▶); data reduction: *SAINT-Plus*; program(s) used to solve structure: *SHELXS97* (Sheldrick, 2008 ▶); program(s) used to refine structure: *SHELXL97* (Sheldrick, 2008 ▶); molecular graphics: *ORTEP-3* (Farrugia, 1997 ▶) and *CAMERON* (Watkin *et al.*, 1996 ▶); software used to prepare material for publication: *WinGX* (Farrugia, 1999 ▶).

## Supplementary Material

Crystal structure: contains datablock(s) global, I. DOI: 10.1107/S1600536812012354/pv2518sup1.cif

Structure factors: contains datablock(s) I. DOI: 10.1107/S1600536812012354/pv2518Isup2.hkl

Supplementary material file. DOI: 10.1107/S1600536812012354/pv2518Isup3.cml

Additional supplementary materials:  crystallographic information; 3D view; checkCIF report

## Figures and Tables

**Table 1:** Hydrogen-bond geometry (Å, °)

*D*—H⋯*A*	*D*—H	H⋯*A*	*D*⋯*A*	*D*—H⋯*A*
C24—H24*C*⋯O5^i^	0.96	2.63	3.537 (3)	158
C25—H25*A*⋯O5^i^	0.96	2.57	3.319 (3)	135

Symmetry code: (i) 
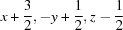
.

**Fig. 1.**
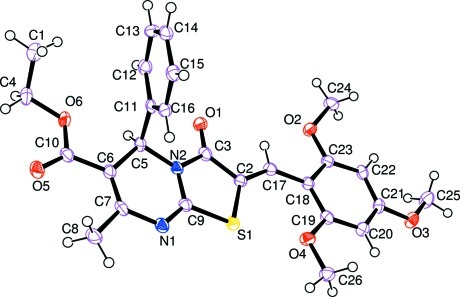
ORTEP (Farrugia, 1997) view of the title compound, showing 50% probability ellipsoids and the atom numbering scheme.

**Fig. 2.**
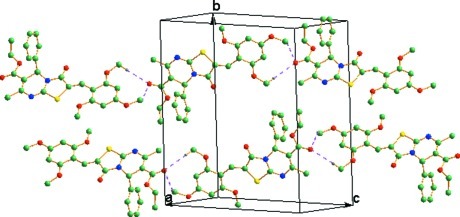
A unit cell packing of the title compound showing intermolecular interactions with dotted lines. H-atoms not involved in hydrogen bonding have been excluded for clarity.

## supplementary crystallographic information

### Comment

Pyrimidine derivatives are of interest because of their pharmacological properties (Alam *et al.*, 2010). In the title compound (Fig. 1), the central pyrimidine ring with a chiral C5 atom is significantly puckered and adopts conformation as seen earlier (Chen *et al.*, 2012). The atom C5 deviates from the mean plane formed by the atoms N2/C9/N1/C6/C7 by 0.224 (2) Å, indicating that the conformation of the ring is that of a flattened boat. In the molecule, the fused thiazolopyrimidine ring makes a dihedral angle of 80.94 (7)° with the benzene ring (C11–C16). In the crystal, pairs of C—H···O hydrogen bonds are bifurcated linking the molecules into chains along *c*-axis (Fig. 2 and Table 1).

### Experimental

A mixture of 5-phenyl-6-methyl-2-thioxo-1,2,3,4-tetrahydro -pyrimidine-5-carboxylic acid ethyl ester (0.01 mol, 2.76 g), chloroaceticacid (0.01 mol, 0.94 g), 2,4,6-trimethoxy benzaldehyde (0.01 mol, 1.96 g) and sodium acetate (1.5 g) in a mixture of glacial acetic acid and acetic anhydride (25 ml, 1:1) was refluxed for 8–10 h. The reaction mixture was concentrated and the solid thus obtained was filtered and recrystallized from ethyl acetate to get the title compound (78% yield, mp 427–428 K). The compound was recrystallized by slow evaporation of an ethyl acetate-ethanol (3:2) solution, yielding pale yellow single crystals suitable for X-ray diffraction studies.

### Refinement

The H atoms were placed at calculated positions in the riding model approximation with C—H = 0.93, 0.96 and 0.98 Å for aryl, methyl and methylene H-atoms, respectively, with *U*_iso_(H) = 1.2–1.5*U*_eq_(C).

### Crystal data

**Table:**

C_26_H_26_N_2_O_6_S	*F*(000) = 1040
*M**_r_* = 494.55	*D*_x_ = 1.417 Mg m^−^^3^
Monoclinic, *P*2_1_/*n*	Mo *K*α radiation, λ = 0.71073 Å
Hall symbol: -P 2yn	Cell parameters from 5055 reflections
*a* = 7.5363 (19) Å	θ = 2.2–27.0°
*b* = 18.178 (5) Å	µ = 0.19 mm^−^^1^
*c* = 16.973 (4) Å	*T* = 296 K
β = 94.465 (5)°	Block, yellow
*V* = 2318.1 (10) Å^3^	0.18 × 0.16 × 0.16 mm
*Z* = 4	

### Data collection

**Table:**

Bruker SMART APEX CCD detector diffractometer	5055 independent reflections
Radiation source: fine-focus sealed tube	3500 reflections with *I* > 2σ(*I*)
Graphite monochromator	*R*_int_ = 0.052
ω scans	θ_max_ = 27.0°, θ_min_ = 2.2°
Absorption correction: multi-scan (*SADABS*; Bruker, 1998)	*h* = −9→6
*T*_min_ = 0.967, *T*_max_ = 0.971	*k* = −19→23
13918 measured reflections	*l* = −21→20

### Refinement

**Table:**

Refinement on *F*^2^	Primary atom site location: structure-invariant direct methods
Least-squares matrix: full	Secondary atom site location: difference Fourier map
*R*[*F*^2^ > 2σ(*F*^2^)] = 0.058	Hydrogen site location: inferred from neighbouring sites
*wR*(*F*^2^) = 0.168	H-atom parameters constrained
*S* = 1.07	*w* = 1/[σ^2^(*F*_o_^2^) + (0.0854*P*)^2^] where *P* = (*F*_o_^2^ + 2*F*_c_^2^)/3
5055 reflections	(Δ/σ)_max_ = 0.001
321 parameters	Δρ_max_ = 0.59 e Å^−^^3^
0 restraints	Δρ_min_ = −0.38 e Å^−^^3^

### Special details

**Table:**

Geometry. All s.u.'s (except the s.u. in the dihedral angle between two l.s. planes) are estimated using the full covariance matrix. The cell s.u.'s are taken into account individually in the estimation of s.u.'s in distances, angles and torsion angles; correlations between s.u.'s in cell parameters are only used when they are defined by crystal symmetry. An approximate (isotropic) treatment of cell s.u.'s is used for estimating s.u.'s involving l.s. planes.
Refinement. Refinement of *F*^2^ against ALL reflections. The weighted *R*-factor *wR* and goodness of fit *S* are based on *F*^2^, conventional *R*-factors *R* are based on *F*, with *F* set to zero for negative *F*^2^. The threshold expression of *F*^2^ > 2σ(*F*^2^) is used only for calculating *R*-factors(gt) *etc*. and is not relevant to the choice of reflections for refinement. *R*-factors based on *F*^2^ are statistically about twice as large as those based on *F*, and *R*- factors based on ALL data will be even larger.

### Fractional atomic coordinates and isotropic or equivalent isotropic displacement parameters (Å^2^)

**Table:**

	*x*	*y*	*z*	*U*_iso_*/*U*_eq_	
S1	0.73105 (9)	0.14251 (3)	0.58179 (4)	0.02186 (19)	
O5	−0.0478 (3)	0.28751 (10)	0.67802 (11)	0.0280 (5)	
O2	1.1137 (2)	0.27911 (9)	0.35791 (11)	0.0259 (5)	
O4	1.0502 (2)	0.09339 (9)	0.54509 (10)	0.0250 (4)	
N1	0.4401 (3)	0.16361 (12)	0.65853 (12)	0.0224 (5)	
O1	0.6191 (2)	0.32009 (9)	0.46396 (11)	0.0252 (4)	
O6	0.0574 (2)	0.36348 (10)	0.58753 (12)	0.0285 (5)	
N2	0.5119 (3)	0.25231 (11)	0.56348 (12)	0.0209 (5)	
O3	1.5463 (3)	0.08397 (10)	0.39073 (11)	0.0276 (5)	
C16	0.5164 (3)	0.36754 (14)	0.69908 (15)	0.0218 (6)	
H16	0.5270	0.3219	0.7237	0.026*	
C11	0.4425 (3)	0.37259 (14)	0.62157 (15)	0.0195 (6)	
C6	0.2376 (4)	0.26532 (14)	0.62849 (15)	0.0220 (6)	
C10	0.0685 (4)	0.30403 (14)	0.63572 (16)	0.0237 (6)	
C20	1.3047 (4)	0.08844 (14)	0.46749 (15)	0.0221 (6)	
H20	1.3473	0.0459	0.4929	0.027*	
C9	0.5401 (4)	0.18888 (13)	0.60625 (15)	0.0207 (6)	
C17	0.9132 (3)	0.22160 (13)	0.46628 (15)	0.0201 (6)	
H17	0.8968	0.2644	0.4365	0.024*	
C7	0.2822 (4)	0.20292 (14)	0.66861 (15)	0.0221 (6)	
C2	0.7777 (3)	0.21079 (13)	0.51239 (15)	0.0209 (6)	
C5	0.3682 (3)	0.30400 (13)	0.57885 (16)	0.0214 (6)	
H5	0.3062	0.3186	0.5284	0.026*	
C3	0.6336 (4)	0.26750 (14)	0.50774 (15)	0.0218 (6)	
C18	1.0776 (3)	0.18488 (13)	0.45027 (15)	0.0204 (6)	
C21	1.3967 (3)	0.12094 (14)	0.40848 (15)	0.0220 (6)	
C8	0.1730 (4)	0.16755 (15)	0.72924 (16)	0.0288 (7)	
H8A	0.1686	0.1997	0.7739	0.043*	
H8B	0.2265	0.1217	0.7460	0.043*	
H8C	0.0544	0.1588	0.7063	0.043*	
C19	1.1482 (4)	0.12100 (14)	0.48744 (14)	0.0206 (6)	
C15	0.5743 (4)	0.43039 (14)	0.73975 (16)	0.0236 (6)	
H15	0.6251	0.4268	0.7913	0.028*	
C12	0.4283 (4)	0.44127 (14)	0.58605 (16)	0.0242 (6)	
H12	0.3805	0.4452	0.5340	0.029*	
C22	1.3384 (3)	0.18520 (14)	0.37125 (15)	0.0213 (6)	
H22	1.4033	0.2070	0.3331	0.026*	
C13	0.4841 (4)	0.50407 (14)	0.62696 (17)	0.0261 (6)	
H13	0.4728	0.5499	0.6027	0.031*	
C23	1.1808 (4)	0.21644 (13)	0.39216 (15)	0.0216 (6)	
C25	1.6375 (4)	0.11104 (16)	0.32485 (17)	0.0328 (7)	
H25A	1.5561	0.1127	0.2785	0.049*	
H25B	1.7348	0.0789	0.3157	0.049*	
H25C	1.6822	0.1596	0.3366	0.049*	
C14	0.5563 (4)	0.49832 (14)	0.70364 (16)	0.0247 (6)	
H14	0.5934	0.5404	0.7313	0.030*	
C26	1.1201 (4)	0.03154 (14)	0.58899 (15)	0.0255 (6)	
H26A	1.1362	−0.0087	0.5536	0.038*	
H26B	1.0386	0.0173	0.6270	0.038*	
H26C	1.2325	0.0444	0.6159	0.038*	
C24	1.2289 (4)	0.32127 (14)	0.31204 (16)	0.0267 (6)	
H24A	1.3356	0.3335	0.3440	0.040*	
H24B	1.1695	0.3657	0.2942	0.040*	
H24C	1.2587	0.2929	0.2672	0.040*	
C1	−0.0260 (4)	0.48913 (16)	0.6104 (2)	0.0390 (8)	
H1A	0.0262	0.4898	0.6638	0.059*	
H1B	−0.1234	0.5233	0.6052	0.059*	
H1C	0.0620	0.5030	0.5752	0.059*	
C4	−0.0928 (4)	0.41300 (15)	0.59001 (19)	0.0325 (7)	
H4A	−0.1703	0.3963	0.6294	0.039*	
H4B	−0.1606	0.4136	0.5390	0.039*	

### Atomic displacement parameters (Å^2^)

**Table:**

	*U*^11^	*U*^22^	*U*^33^	*U*^12^	*U*^13^	*U*^23^
S1	0.0216 (4)	0.0219 (4)	0.0231 (4)	−0.0004 (3)	0.0078 (3)	0.0010 (3)
O5	0.0244 (11)	0.0318 (11)	0.0296 (11)	−0.0015 (9)	0.0135 (9)	−0.0016 (8)
O2	0.0233 (11)	0.0237 (10)	0.0322 (11)	0.0035 (8)	0.0118 (8)	0.0075 (8)
O4	0.0284 (11)	0.0222 (10)	0.0262 (10)	0.0018 (8)	0.0128 (8)	0.0051 (7)
N1	0.0211 (13)	0.0240 (11)	0.0229 (12)	−0.0026 (10)	0.0072 (10)	−0.0004 (9)
O1	0.0235 (11)	0.0232 (10)	0.0299 (11)	0.0014 (8)	0.0095 (8)	0.0031 (8)
O6	0.0181 (10)	0.0291 (10)	0.0396 (12)	0.0037 (8)	0.0111 (9)	0.0051 (8)
N2	0.0177 (12)	0.0211 (11)	0.0250 (12)	−0.0024 (9)	0.0081 (9)	−0.0007 (9)
O3	0.0245 (11)	0.0334 (11)	0.0265 (10)	0.0095 (9)	0.0123 (8)	0.0050 (8)
C16	0.0200 (14)	0.0208 (13)	0.0256 (14)	0.0003 (11)	0.0082 (11)	0.0020 (10)
C11	0.0128 (13)	0.0232 (13)	0.0239 (14)	−0.0011 (10)	0.0098 (11)	−0.0023 (10)
C6	0.0184 (14)	0.0207 (13)	0.0275 (14)	−0.0013 (11)	0.0064 (11)	−0.0046 (11)
C10	0.0211 (15)	0.0229 (14)	0.0277 (15)	−0.0056 (11)	0.0059 (12)	−0.0064 (11)
C20	0.0225 (15)	0.0243 (13)	0.0196 (13)	0.0023 (11)	0.0016 (11)	0.0010 (10)
C9	0.0201 (14)	0.0191 (13)	0.0232 (14)	−0.0026 (11)	0.0038 (11)	−0.0048 (10)
C17	0.0221 (15)	0.0196 (13)	0.0194 (13)	−0.0020 (11)	0.0062 (11)	−0.0012 (10)
C7	0.0189 (14)	0.0244 (14)	0.0237 (14)	−0.0079 (11)	0.0065 (11)	−0.0064 (11)
C2	0.0201 (14)	0.0184 (13)	0.0246 (14)	−0.0011 (11)	0.0050 (11)	−0.0018 (10)
C5	0.0159 (14)	0.0240 (13)	0.0251 (14)	0.0008 (11)	0.0072 (11)	−0.0012 (11)
C3	0.0207 (15)	0.0243 (14)	0.0211 (14)	−0.0032 (11)	0.0066 (11)	−0.0026 (11)
C18	0.0190 (14)	0.0204 (13)	0.0227 (14)	−0.0009 (11)	0.0069 (11)	−0.0019 (10)
C21	0.0173 (14)	0.0273 (14)	0.0215 (13)	0.0023 (11)	0.0029 (11)	−0.0024 (11)
C8	0.0292 (17)	0.0258 (14)	0.0334 (16)	−0.0049 (12)	0.0143 (13)	−0.0007 (12)
C19	0.0215 (14)	0.0233 (13)	0.0177 (13)	−0.0036 (11)	0.0064 (11)	−0.0015 (10)
C15	0.0204 (14)	0.0284 (14)	0.0228 (14)	0.0002 (12)	0.0060 (11)	−0.0034 (11)
C12	0.0212 (15)	0.0286 (14)	0.0234 (14)	0.0015 (11)	0.0057 (11)	0.0042 (11)
C22	0.0173 (14)	0.0282 (14)	0.0191 (13)	−0.0023 (11)	0.0059 (11)	−0.0022 (10)
C13	0.0258 (16)	0.0161 (13)	0.0376 (17)	0.0012 (11)	0.0095 (13)	0.0043 (11)
C23	0.0215 (15)	0.0187 (13)	0.0252 (14)	−0.0018 (11)	0.0051 (11)	−0.0014 (10)
C25	0.0297 (17)	0.0396 (17)	0.0311 (16)	0.0119 (14)	0.0146 (13)	0.0078 (13)
C14	0.0207 (15)	0.0222 (14)	0.0323 (15)	−0.0028 (11)	0.0096 (12)	−0.0053 (11)
C26	0.0276 (16)	0.0230 (14)	0.0265 (15)	0.0008 (12)	0.0063 (12)	0.0031 (11)
C24	0.0296 (16)	0.0240 (14)	0.0276 (15)	−0.0031 (12)	0.0091 (13)	0.0065 (11)
C1	0.0296 (18)	0.0357 (17)	0.053 (2)	0.0054 (14)	0.0103 (15)	0.0013 (15)
C4	0.0186 (15)	0.0355 (16)	0.0447 (18)	0.0046 (13)	0.0103 (13)	0.0040 (13)

### Geometric parameters (Å, º)

**Table:**

S1—C9	1.745 (3)	C2—C3	1.495 (4)
S1—C2	1.765 (3)	C5—H5	0.9800
O5—C10	1.213 (3)	C18—C19	1.406 (4)
O2—C23	1.359 (3)	C18—C23	1.423 (3)
O2—C24	1.433 (3)	C21—C22	1.383 (4)
O4—C19	1.367 (3)	C8—H8A	0.9600
O4—C26	1.427 (3)	C8—H8B	0.9600
N1—C9	1.292 (3)	C8—H8C	0.9600
N1—C7	1.410 (3)	C15—C14	1.381 (4)
O1—C3	1.211 (3)	C15—H15	0.9300
O6—C10	1.354 (3)	C12—C13	1.385 (4)
O6—C4	1.449 (3)	C12—H12	0.9300
N2—C9	1.370 (3)	C22—C23	1.387 (3)
N2—C3	1.395 (3)	C22—H22	0.9300
N2—C5	1.473 (3)	C13—C14	1.375 (4)
O3—C21	1.367 (3)	C13—H13	0.9300
O3—C25	1.443 (3)	C25—H25A	0.9600
C16—C15	1.387 (4)	C25—H25B	0.9600
C16—C11	1.391 (4)	C25—H25C	0.9600
C16—H16	0.9300	C14—H14	0.9300
C11—C12	1.387 (3)	C26—H26A	0.9600
C11—C5	1.527 (3)	C26—H26B	0.9600
C6—C7	1.352 (4)	C26—H26C	0.9600
C6—C10	1.469 (4)	C24—H24A	0.9600
C6—C5	1.517 (3)	C24—H24B	0.9600
C20—C19	1.385 (3)	C24—H24C	0.9600
C20—C21	1.393 (3)	C1—C4	1.504 (4)
C20—H20	0.9300	C1—H1A	0.9600
C17—C2	1.348 (3)	C1—H1B	0.9600
C17—C18	1.451 (3)	C1—H1C	0.9600
C17—H17	0.9300	C4—H4A	0.9700
C7—C8	1.511 (3)	C4—H4B	0.9700
			
C9—S1—C2	91.72 (12)	C7—C8—H8C	109.5
C23—O2—C24	117.4 (2)	H8A—C8—H8C	109.5
C19—O4—C26	117.7 (2)	H8B—C8—H8C	109.5
C9—N1—C7	116.5 (2)	O4—C19—C20	122.4 (2)
C10—O6—C4	119.3 (2)	O4—C19—C18	114.7 (2)
C9—N2—C3	116.3 (2)	C20—C19—C18	122.9 (2)
C9—N2—C5	121.9 (2)	C14—C15—C16	120.0 (3)
C3—N2—C5	121.7 (2)	C14—C15—H15	120.0
C21—O3—C25	117.0 (2)	C16—C15—H15	120.0
C15—C16—C11	120.2 (2)	C13—C12—C11	120.9 (3)
C15—C16—H16	119.9	C13—C12—H12	119.5
C11—C16—H16	119.9	C11—C12—H12	119.5
C12—C11—C16	118.8 (2)	C21—C22—C23	118.5 (2)
C12—C11—C5	121.0 (2)	C21—C22—H22	120.8
C16—C11—C5	120.0 (2)	C23—C22—H22	120.8
C7—C6—C10	122.9 (2)	C14—C13—C12	119.6 (2)
C7—C6—C5	121.5 (2)	C14—C13—H13	120.2
C10—C6—C5	115.4 (2)	C12—C13—H13	120.2
O5—C10—O6	122.8 (2)	O2—C23—C22	122.1 (2)
O5—C10—C6	127.0 (3)	O2—C23—C18	115.4 (2)
O6—C10—C6	110.2 (2)	C22—C23—C18	122.5 (2)
C19—C20—C21	118.4 (2)	O3—C25—H25A	109.5
C19—C20—H20	120.8	O3—C25—H25B	109.5
C21—C20—H20	120.8	H25A—C25—H25B	109.5
N1—C9—N2	126.0 (2)	O3—C25—H25C	109.5
N1—C9—S1	121.8 (2)	H25A—C25—H25C	109.5
N2—C9—S1	112.16 (18)	H25B—C25—H25C	109.5
C2—C17—C18	137.5 (2)	C13—C14—C15	120.4 (2)
C2—C17—H17	111.2	C13—C14—H14	119.8
C18—C17—H17	111.2	C15—C14—H14	119.8
C6—C7—N1	122.9 (2)	O4—C26—H26A	109.5
C6—C7—C8	124.9 (2)	O4—C26—H26B	109.5
N1—C7—C8	112.1 (2)	H26A—C26—H26B	109.5
C17—C2—C3	116.7 (2)	O4—C26—H26C	109.5
C17—C2—S1	133.6 (2)	H26A—C26—H26C	109.5
C3—C2—S1	109.77 (18)	H26B—C26—H26C	109.5
N2—C5—C6	108.7 (2)	O2—C24—H24A	109.5
N2—C5—C11	110.9 (2)	O2—C24—H24B	109.5
C6—C5—C11	110.2 (2)	H24A—C24—H24B	109.5
N2—C5—H5	109.0	O2—C24—H24C	109.5
C6—C5—H5	109.0	H24A—C24—H24C	109.5
C11—C5—H5	109.0	H24B—C24—H24C	109.5
O1—C3—N2	122.7 (2)	C4—C1—H1A	109.5
O1—C3—C2	127.4 (2)	C4—C1—H1B	109.5
N2—C3—C2	109.9 (2)	H1A—C1—H1B	109.5
C19—C18—C23	115.8 (2)	C4—C1—H1C	109.5
C19—C18—C17	126.5 (2)	H1A—C1—H1C	109.5
C23—C18—C17	117.7 (2)	H1B—C1—H1C	109.5
O3—C21—C22	123.6 (2)	O6—C4—C1	109.3 (2)
O3—C21—C20	114.5 (2)	O6—C4—H4A	109.8
C22—C21—C20	121.9 (2)	C1—C4—H4A	109.8
C7—C8—H8A	109.5	O6—C4—H4B	109.8
C7—C8—H8B	109.5	C1—C4—H4B	109.8
H8A—C8—H8B	109.5	H4A—C4—H4B	108.3
			
C15—C16—C11—C12	0.1 (4)	C5—N2—C3—O1	−6.9 (4)
C15—C16—C11—C5	−176.1 (2)	C9—N2—C3—C2	−3.8 (3)
C4—O6—C10—O5	4.6 (4)	C5—N2—C3—C2	172.4 (2)
C4—O6—C10—C6	−175.1 (2)	C17—C2—C3—O1	4.5 (4)
C7—C6—C10—O5	1.9 (4)	S1—C2—C3—O1	−176.6 (2)
C5—C6—C10—O5	−172.4 (3)	C17—C2—C3—N2	−174.8 (2)
C7—C6—C10—O6	−178.4 (2)	S1—C2—C3—N2	4.1 (3)
C5—C6—C10—O6	7.3 (3)	C2—C17—C18—C19	−3.3 (5)
C7—N1—C9—N2	4.1 (4)	C2—C17—C18—C23	177.7 (3)
C7—N1—C9—S1	−175.11 (18)	C25—O3—C21—C22	−5.5 (4)
C3—N2—C9—N1	−177.5 (2)	C25—O3—C21—C20	174.1 (2)
C5—N2—C9—N1	6.3 (4)	C19—C20—C21—O3	−178.0 (2)
C3—N2—C9—S1	1.8 (3)	C19—C20—C21—C22	1.7 (4)
C5—N2—C9—S1	−174.44 (18)	C26—O4—C19—C20	4.1 (4)
C2—S1—C9—N1	180.0 (2)	C26—O4—C19—C18	−175.8 (2)
C2—S1—C9—N2	0.7 (2)	C21—C20—C19—O4	−179.4 (2)
C10—C6—C7—N1	176.5 (2)	C21—C20—C19—C18	0.6 (4)
C5—C6—C7—N1	−9.6 (4)	C23—C18—C19—O4	177.6 (2)
C10—C6—C7—C8	−4.2 (4)	C17—C18—C19—O4	−1.4 (4)
C5—C6—C7—C8	169.8 (2)	C23—C18—C19—C20	−2.4 (4)
C9—N1—C7—C6	−2.2 (4)	C17—C18—C19—C20	178.6 (2)
C9—N1—C7—C8	178.3 (2)	C11—C16—C15—C14	0.9 (4)
C18—C17—C2—C3	179.5 (3)	C16—C11—C12—C13	−0.8 (4)
C18—C17—C2—S1	0.9 (5)	C5—C11—C12—C13	175.3 (2)
C9—S1—C2—C17	175.9 (3)	O3—C21—C22—C23	177.6 (2)
C9—S1—C2—C3	−2.7 (2)	C20—C21—C22—C23	−1.9 (4)
C9—N2—C5—C6	−15.9 (3)	C11—C12—C13—C14	0.6 (4)
C3—N2—C5—C6	168.1 (2)	C24—O2—C23—C22	−13.5 (4)
C9—N2—C5—C11	105.4 (3)	C24—O2—C23—C18	167.1 (2)
C3—N2—C5—C11	−70.6 (3)	C21—C22—C23—O2	−179.4 (2)
C7—C6—C5—N2	17.4 (3)	C21—C22—C23—C18	0.0 (4)
C10—C6—C5—N2	−168.2 (2)	C19—C18—C23—O2	−178.5 (2)
C7—C6—C5—C11	−104.3 (3)	C17—C18—C23—O2	0.6 (3)
C10—C6—C5—C11	70.0 (3)	C19—C18—C23—C22	2.0 (4)
C12—C11—C5—N2	115.6 (3)	C17—C18—C23—C22	−178.8 (2)
C16—C11—C5—N2	−68.3 (3)	C12—C13—C14—C15	0.3 (4)
C12—C11—C5—C6	−123.9 (3)	C16—C15—C14—C13	−1.1 (4)
C16—C11—C5—C6	52.1 (3)	C10—O6—C4—C1	121.5 (3)
C9—N2—C3—O1	176.8 (2)		

### Hydrogen-bond geometry (Å, º)

**Table:**

*D*—H···*A*	*D*—H	H···*A*	*D*···*A*	*D*—H···*A*
C24—H24*C*···O5^i^	0.96	2.63	3.537 (3)	158
C25—H25*A*···O5^i^	0.96	2.57	3.319 (3)	135

Symmetry code: (i) *x*+3/2, −*y*+1/2, *z*−1/2.
